# Supplementary material for: The Generation of Promoter-Mediated Transcriptional Noise in Bacteria
Source: PLoS Comput Biol. 2008 Jul 11;4(7):e1000109. doi: 10.1371/journal.pcbi.1000109 (PMC2442219; doi:10.1371/journal.pcbi.1000109)
Supplement: Text S1 — Supplemental materials (0.13 MB PDF) [file pcbi.1000109.s001.pdf]

The generation of promoter-mediated transcriptional noise in bacteria

Namiko Mitarai, Ian B. Dodd, Michael T. Crooks, and Kim Sneppen

Supplement

1. Promoter strength with recruitment

With transition rates defined in figure 2(a) we here present equations that connect these intrinsic promoter parameters to the overall activity and occupation probabilities of the promoter. With η and θ being respectively the probability that the promoter is occupied by a closed or open complex, the steady state implies:

$$k_b(1-\eta-\theta-E\theta\frac{l}{v})=(k_u+O)\eta, \quad (5)$$

$$O\eta+qE\theta=E\theta, \quad (6)$$

which can be solved for η and θ. Note that eq.(2) takes into account the occlusion of the promoter the elongating RNAP as it leaves the promoter region of length l with velocity v. One finds:

$$\theta=\frac{O}{E(1-q)(1+K)+O(1+El/v)+cE(1-q)/k_b}$$

$$=\frac{1}{(1-q)/\alpha+(1+El/v)+E(1-q)/k_b}, \quad (7)$$

where the last equality expresses θ in terms of the aspect ratio

$$\alpha=\frac{O}{(1+K)E}=O'/E \quad (8)$$

introduced by [8], where $O'=O/(1+K)$. $\alpha\approx 1$ corresponds to class II promoters, whereas very large or small α effectively corresponds to a single limiting step in transcription initiation (class I). Recruitment into the open complex will predict bunched activity for $\alpha << 1$ where formation of the open complex is rate limiting.

The promoter strength activity given by the rate $E\theta$

$$F = \frac{EO}{E(1-q)(1+K) + O(1+E\,\frac{l}{v}) + OE(1-q)/k_b} \tag{9}$$

For $q \to 1$ the rate is approximated by:

$$F = \frac{E}{(1+El/v)} \tag{10}$$

This reflects transcription that is governed by elongation initiation, which in turn is limited by self occlusion.

For a promoter where self occlusion is insignificant, the occupation probabilities are simplified to:

$$\eta = \frac{1}{1+K+O/k_b+O/((1-q)E)}, \tag{11}$$

and

$$\theta = \frac{O}{O+(1-q)(K+1)E+(1-q)\cdot E\cdot O/k_b}. \tag{12}$$

In the limit of large k_b, this becomes

$$\theta = \frac{\alpha}{\alpha+(1-q)}. \tag{13}$$

Note that all the equations here recover into the one for the standard three-step model if we set the recruitment probability $q = 0$.

2. Promoter strength with dead-end complexes

We can also develop an expression for the strength of the promoter when a fraction $1-Q$ of complexes enter into a non-productive state, from which the only escape is through the removal of the stalled RNAP with a rate d (Fig. 2(b)). We denote the other rates, k_b, k_u, O and E as before, whereas Ω is the probability that promoter is occluded by a non-productive complex. As before η and θ is respectively the probability that the promoter is occupied by closed and open complex. Steady state equations for occupancy of the promoter are now:

$$k_b(1-\eta-\theta-QE\theta\frac{l}{v}-\Omega) = (k_u+O)\eta \tag{14}$$

$$O\eta = E\theta \tag{15}$$

$$(1-Q)E\theta = d\Omega \tag{16}$$

These are solved to give probabilities for the promoter to be occupied by respectively a productive open complex (θ), a non-productive complex (Ω), or a closed complex (η):

$$\theta = \frac{O}{(1+K)E + OE/k_b + (1+QEl/v)O + (1-Q)OE/d}$$

$$\Omega = \frac{E}{d}(1-Q)\,\theta$$

$$\eta = \frac{E}{O}\theta \tag{17}$$

The promoter strength is then $QE\theta$, or

$$F = \frac{QEO}{(1+K)E + OE/k_b + (1+QEl/v)O + (1-Q)OE/d} \tag{18}$$

For $Q=1$ this corresponds to the activity of a promoter without any dead-end complex (the term containing d disappears). Also when the rate of removing dead-end complexes is very high, $d \to \infty$, the main effect of their presence is a reduction of the effective firing rate from open complexes from E to the lower value QE.

When the self occlusion is negligible and k_b is large compared to other non-equilibrium rates, we get

$$F = \frac{Qd}{\beta + (1-Q)}, \tag{19}$$

Here, $\beta = (\tau_O + \tau_E)/\tau_{dead} = d(E+O')/EO'$ is the ratio of the time needed for successful firing and the time to remove a dead-end complex.

3. Distribution of time intervals between firings

In figure 1bc and figure 3be, we showed the distributions $P(\Delta t)$ of the time interval between firings, Δt. We present the equations for $P(\Delta t)$ for our model. We consider the case where the k_b and k_u are large enough that the binding and the unbinding of closed complexes are considered to be in equilibrium and the process is dominated by two steps, the formation of the open complex with a rate $1/\tau_O = O/(1+K)$ and the formation of the elongating complex with a rate $1/\tau_E = E$.

The distribution for the standard model are eqs.(1) and (2) in the main text, and the calculation is given in Materials and Methods.

In the case with supercoiling assisted recruitment with probability q, the distribution

is simply sum of the distribution for unassisted initiation (eq. (1) in the main text) with a weight $(1-q)$ and the distribution for the single supercoiling assisted step from open complex to elongating complex with a weight q, which is given by

$$P(\Delta t) = q \frac{\exp\left(-\frac{\Delta t}{\tau_E}\right)}{\tau_E} + (1-q) \frac{\left[\exp\left(-\frac{\Delta t}{\tau_O}\right) - \exp\left(-\frac{\Delta t}{\tau_E}\right)\right]}{\tau_O - \tau_E}.$$

In the case $\tau_E << \tau_O$, this is approximated as

$$P(\Delta t) = \frac{q}{\tau_E} \exp\left(-\frac{\Delta t}{\tau_E}\right) + \frac{(1-q)}{\tau_O} \exp\left(-\frac{\Delta t}{\tau_O}\right). \quad (20)$$

Similarly, in the case of the dead-end model, the distribution is approximately the sum of the distribution for successful initiation with a weight Q and the distribution of the silenced periods caused by the dead-end complex with a weight $(1-Q)$ and average $\tau_{dead/Q}$, or

$$P(\Delta t) = Q \frac{\left[\exp\left(-\frac{\Delta t}{\tau_O}\right) - \exp\left(-\frac{\Delta t}{\tau_E}\right)\right]}{\tau_O - \tau_E} + (1-Q) \frac{\exp\left(-\frac{\Delta t}{\tau_{dead}/Q}\right)}{\tau_{dead}/Q}.$$

This estimation does not take into account the time needed to form a dead-end complex, but this effect is negligible when removing the dead-end complex is the rate limiting step, i.e. $\tau_{dead} >> \tau_O + \tau_E$.

4. Distribution of successive firings due to recruitment

The distribution of Δn in the supercoiling assisted recruitment model is determined by the probability to have $\Delta n - 1$ successive recruitments under the condition that *the first firing occurred*[1]. Because the probability of recruitment is q, the probability to

[1]Precisely speaking, this is different from the Δn defined in Section 4.2, because the

have Δn is given by

$$P_{\Delta n} = q^{\Delta n-1} \cdot (1-q). \tag{21}$$

The average number of events is given by

$$\langle \Delta n \rangle = \sum_{\Delta n=1}^{\infty} \Delta n P_{\Delta n} = (1-q) \sum_{\Delta n=1}^{\infty} \Delta n q^{\Delta n-1} = \frac{1}{1-q}. \tag{22}$$

5. On-time distribution and average for the recruitment model

Here we calculate the probability for the on-time to be t_{on}, considering that the on-time is the duration when a transcription event occurs and then possibly supercoiling assisted recruitment into open complexes occurs successively. First, we calculate on period times for the case when the duration to transcribe one message, Δ, is zero, which means that on-time is given by the sum of the intervals between successive transcription events. (Thus events of $\Delta n = 1$ do not contribute.) The duration between events obeys the exponential distribution $\exp(-t/\tau_E)/\tau_E$.

The probability to have n (≥ 2) successive events giving t_{on} is proportional to:

$$P_n(t_{on}) = (1-q) q^{n-1} \frac{1}{\tau_E^{n-1}} \times \int_{\sum_{i=1}^{n-1} t_i = t_{on}} [\prod_{i=1}^{n-2} dt_i][\prod_{i=1}^{n-1} e^{-t_i/\tau_E}] \tag{23}$$

$$= (1-q) q^{n-1} \frac{1}{\tau_E^{n-1}} e^{-t_{on}/\tau_E} \times \int_{\sum_{i=1}^{n-1} t_i = t_{on}} \prod_{i=1}^{n-2} dt_i. \tag{24}$$

The integral means the volume defined by $\sum_{i=1}^{n-1} t_i = t_{on}$ with $t_i > 0$, given by

$$\int_0^{t_{on}} ds_1 \int_0^{s_1} ds_2 \int_0^{s_2} ds_3 \cdots \int_0^{s_{n-3}} ds_{n-2} = \frac{1}{(n-2)!} t_{on}^{n-2}. \tag{25}$$

Finally we get

---

probability that the duration between successive firing with recruitment being longer than the threshold τ_c is not zero. However, this probability is so small in the present parameter regime that the difference does not matter in practice.

$$P_n(t_{on}) = (1-q)q^{n-1}\frac{1}{\tau_E^{n-1}}\frac{1}{(n-2)!}t_{on}^{n-2}e^{-t_{on}/\tau_E}. \quad (26)$$

Thus, the probability to have on-time t_{on} is

$$P(t_{on}) = (1-q)\delta(t_{on}) + (1-q)\frac{q}{\tau_E}e^{-t_{on}/\tau_E}$$

$$\times\sum_{n=2}^{\infty}\frac{1}{(n-2)!}\left(\frac{qt_{on}}{\tau_E}\right)^{n-2} \quad (27)$$

$$= (1-q)\delta(t_{on}) + (1-q)\frac{q}{\tau_E}$$
$$\times e^{-t_{on}/\tau_E}e^{qt_{on}/\tau_E}, \quad (28)$$

for $0 \le t_{on} < \infty$[2]. Here the δ function takes into account that a single event is counted with duration $t_{on} = 0$, and with probability $1-q$ (given that we already started with this single event).

Using $q = 1 - 1/\langle \Delta n \rangle$ one for $t_{on} > 0$ get the on-time distribution

$$P(t_{on}) = \frac{\langle \Delta n \rangle - 1}{\langle \Delta n \rangle}\cdot\frac{1}{\langle \Delta n \rangle \tau_E}e^{-t_{on}/(\langle \Delta n \rangle \tau_E)}. \quad (29)$$

The average on-time is given by $\tau_E(\langle \Delta n \rangle - 1)$, which is the duration of the firing from the open complex multiplied by the average number of the successive recruitment events.

1. On-time distribution and average with contribution from events with $\Delta n = 1$.

Now we simply assume that one event gives a fixed on-time Δ. This gives an offset of Δ for the on-time, thus we have

$$P(t_{on}) = (1-q)\delta(t_{on} - \Delta)$$
$$+\frac{(1-q)q}{\tau_E}e^{-(t_{on}-\Delta)/(\langle \Delta n \rangle \tau_E)}. \quad (30)$$

---

[2]Here $\int_0^\infty \delta(t)dt = 1$, not a half.

The average is given by

$$\begin{aligned}\langle t_{on}\rangle &= q(\tau_E/(1-q)+\Delta)+(1-q)\Delta \\ &= q\tau_E/(1-q)+\Delta \\ &= (\langle \Delta n\rangle -1)\tau_E+\Delta. \end{aligned} \tag{31}$$

2. Determination of the parameters.

In the experiments by Golding *et al.*, they obtained the average number of transcriptions per burst, $\langle \Delta n\rangle = 2.2$, the average on-time $\langle t_{on}\rangle = 6$ [min] and the average off-time $\langle t_{off}\rangle = 37$ [min]. They also mention that the duration of transcribing 1 message is $\Delta = 2.5$ [min].

The value of q is determined from $\langle \Delta n\rangle$ using eq. (22) as $q = 1-1/\langle \Delta n\rangle$. The duration from open complex to the elongation complex τ_E is determined by eq. (31) using the average on-time $\langle t_{on}\rangle$. The average off-time $\langle t_{off}\rangle$. is given by $\tau_E+\tau_O-\Delta$, which fixes the time to form the open complex, τ_O.

It should be noted that the on-time distribution given in (30) has a sharp peak at Δ. In the simulation data, the frequency are calculated using the bin with width 5[min] as in the experiment by Golding *et al.*, which makes this peak low.

1. The on-time and off-time distribution in the dead-end complex model

The distribution of Δn and the on-time distribution $P(t_{on})$ in the dead-end model (Fig. 2(b)) are given by similar calculations as the recruitment model: During an on-time the RNAPs take the standard 3-step firing pathway, which takes the time $\tau_O+\tau_E$ per firing, and the probability to take this pathway is Q. In particular, if the distribution of Δt for the full 3-step firing is a single exponential as in the Class 1 case of the standard model, we can simply get $P(t_{on})$ in the dead-end model by replacing the probability of the recruitment q with the probability to take the firing pathway Q and τ_E with $\tau_O+\tau_E$.

As a result, we get the following distribution for the number of transcripts per on-time

$$P_{\Delta n} = 1 \cdot Q^{\Delta n - 1} \cdot (1-Q). \tag{32}$$

The average number of events is

$$\langle \Delta n \rangle = \frac{1}{1-Q}. \tag{33}$$

The distribution of the on-time is given by

$$P(t_{on}) = (1-Q)\delta(t_{on} - \Delta) + \\ (1-Q)\frac{Q}{\tau_O + \tau_E} \times e^{-(t_{on}-\Delta)/(\langle \Delta n \rangle (\tau_O + \tau_E))}. \tag{34}$$

To get the distribution of the off-time, we also need a similar calculation, since if several RNAPs end up in a dead-end complex in a row it makes the off-time longer. When we consider the time spent occupied by a dead-end complex as the off-time, the calculation is parallel to the on-time distribution calculation without Δ in the recruitment model, replacing q with $(1-Q)$ and τ_E with τ_{dead}. The only difference is that the first dead-end event also gives the off-time τ_{dead}.

As a result, the probability to have dead-end complexes n times in a row is given by

$$p_n = Q(1-Q)^{n-1}, \tag{35}$$

which gives the average number of the dead-end complexes in a row as $1/Q$. The off-time distribution is given by a single exponential distribution

$$P(t_{off}) = \frac{1}{\tau_{dead}/Q} e^{-t_{off}/(\tau_{dead}/Q)}. \tag{36}$$

Note that the off-time measured from the experiment can be slightly longer than this, because the calculation here does not include either the duration from the last firing to the first formation of the dead-end complex or the duration from the moment of removal of the last dead-end complex to the next initiation.

2. Calculation of the Fano factor

When mRNAs are degraded with a rate γ, the number of mRNAs reaches a steady state. We calculate the Fano factor for the number of mRNAs by using the Fokker-Plank (FP) equations for both the recruitment model and the dead-end model. We again ignore the effect of occlusion of promoters by an elongating complex.

1. The recruitment model

In the recruitment model, the promoter can take the following 3 states:

- No RNAP at the promoter,
- Closed complex at the promoter,
- Open complex at the promoter.

When the RNAP starts elongation, the promoter goes from state 3 to state 1, with one more mRNA in the system when no recruitment occurs, while the promoter goes back to the state 3 with one more mRNA in the system when the recruitment occurs.

We define the probability $f_i(n;t)$ with $i=1,2,3$ as the probability to have n mRNAs in the system *and* that the promoter takes the state i at time t. The FP equations for $f_i(n;t)$ are given as follows:

$$\frac{df_1(n;t)}{dt} = -k_b f_1(n;t) + k_u f_2(n;t) + (1-q)Ef_3(n-1;t) - \gamma\left[nf_1(n;t) - (n+1) f_1(n+1;t)\right], \tag{37}$$

$$\frac{df_2(n;t)}{dt} = k_b f_1(n;t) - k_u f_2(n;t) - Of_2(n;t) - \gamma\left[nf_2(n;t) - (n+1) f_2(n+1;t)\right], \tag{38}$$

$$\frac{df_3(n;t)}{dt} = Of_2(n;t) + qEf_3(n-1;t) - Ef_3(n;t) - \gamma\left[nf_3(n;t) - (n+1) f_3(n+1;t)\right]. \tag{39}$$

The probability is normalized so that $\sum_{i=1}^{3}\sum_{n=0}^{\infty} f_i(n;t) = 1$. The average number of mRNAs $\langle N\rangle$ and variance $\langle \delta N^2\rangle = \langle (N-\langle N\rangle)^2\rangle$ are given by

$$\langle N\rangle = \sum_{i=1}^{3}\sum_{n=0}^{\infty} nf_i(n;t), \tag{40}$$

$$\langle \delta N^2\rangle = \langle N^2\rangle - \langle N\rangle^2 = \sum_{i=1}^{3}\sum_{n=0}^{\infty} n^2 f_i(n;t) - \langle N\rangle^2, \tag{41}$$

respectively, and the Fano factor is given by $\nu = \langle \delta N^2\rangle / \langle N\rangle$.

In order to calculate them using FP equations, we define the generating functions

$$F_i(z;t) = \sum_{n=0}^{\infty} z^n f_i(n;t). \tag{42}$$

The moments are obtained from

$$1 = \sum_i F_i(1;t), \tag{43}$$

$$\langle N \rangle = \sum_i \frac{dF_i(z;t)}{dz}|_{z=1}, \tag{44}$$

$$\langle N(N-1) \rangle = \sum_i \frac{d^2F_i(z;t)}{dz^2}|_{z=1} . \tag{45}$$

Multiplying eqs.(37)-(39) by z^n and taking summation of n from zero to infinity, we get the equations for the generating function $F_i(z;t)$. Using the derivatives of the equations and the normalization condition (43) in the steady state (i.e. $\frac{d}{dt} f_i(z;t) = 0$), we can calculate the moments. As a result, we get

$$\langle N \rangle = \frac{E}{\gamma} \frac{O'}{O' + (1-q)E(1 + O'/k_b)}, \tag{46}$$

$$\frac{\langle \delta N^2 \rangle}{\langle N \rangle} = 1 - \langle N \rangle + \frac{E}{\gamma} \cdot \frac{k_b O + \gamma q(\gamma + k_u + k_b + O)}{(\gamma + k_u + k_b + O)(E + \gamma - qE) + k_b O}. \tag{47}$$

If equilibrium binding and unbinding are fast enough, i.e, $k_b, k_u >> O, E, \gamma$, we get

$$\langle N \rangle = \frac{E}{\gamma} \frac{O'}{O' + (1-q)E} = \frac{E}{\gamma} \frac{\alpha}{1 - q + \alpha}, \tag{48}$$

$$\nu = \frac{\langle \delta N^2 \rangle}{\langle N \rangle} = 1 + \frac{(1-q)(q-\alpha)}{(\alpha + (1-q))[\alpha + (1-q) + \gamma/E]}, \tag{49}$$

where we used the aspect ration $\alpha = O'/E$. Equation (49) is shown in figure 4(a). In case $\alpha << q$ and $\alpha << 1-q$, we get

$$\langle N \rangle = \frac{E\alpha}{\gamma(1-q)}, \tag{50}$$

$$\nu = \frac{\langle \delta N^2 \rangle}{\langle N \rangle} = 1 + \frac{q}{(1-q) + \gamma/E}, \tag{51}$$

and the recruitment model gives larger fluctuations than a simple Poisson process.

2. The dead-end model

The promoter in the dead-end model can take the following 4 states:

- No RNAP at the promoter,

- Closed complex at the promoter,
- Open complex at the promoter.
- Dead-end complex at the promoter,

We define the probability $f_i(n;t)$ with $i=1,2,3,4$ as the probability to have n mRNAs in the system AND the that promoter takes the state i at time t. The FP equations for $f_i(n;t)$ are given as follows:

$$\frac{df_1(n;t)}{dt} = -k_b f_1(n;t) + k_u f_2(n;t) + QEf_3(n-1;t) + df_4(n;t) - \gamma\left[nf_1(n;t) - (n+1) f_1(n+1;t)\right], \tag{52}$$

$$\frac{df_2(n;t)}{dt} = k_b f_1(n;t) - k_u f_2(n;t) - Of_2(n;t) - \gamma\left[nf_2(n;t) - (n+1) f_2(n+1;t)\right], \tag{53}$$

$$\frac{df_3(n;t)}{dt} = Of_2(n;t) - Ef_3(n;t) - \gamma\left[nf_3(n;t) - (n+1) f_3(n+1;t)\right],, \tag{54}$$

$$\frac{df_4(n;t)}{dt} = (1-Q)Ef_3(n;t) - df_4(n;t) - \gamma\left[nf_4(n;t) - (n+1) f_4(n+1;t)\right]. \tag{55}$$

The probability is normalized so that $\sum_{i=1}^{4}\sum_{n=0}^{\infty} f_i(n;t) = 1$.

By using the generating functions

$$F_i(z;t) = \sum_{n=0}^{\infty} z^n f_i(n;t), \tag{56}$$

and the normalization conditions in the steady state, we get

$$\langle N \rangle = \frac{QE}{\gamma} \frac{\alpha}{\alpha + 1 + O'/k_b + \alpha(1-Q)E/d}, \tag{57}$$

$$\nu = \frac{\langle \delta N^2 \rangle}{\langle N \rangle} = 1 - \frac{QE}{\gamma}\left[\frac{O'}{O' + (1+O'/k_b)E + O'E(1-Q)/d}\right.$$

$$\left. - \frac{O'(d+\gamma)}{(E+\gamma)(d+\gamma) + O'(d+\gamma)(1+(O+\gamma)/(E+\gamma)/(Ok_b)) + (1-Q)EO'}\right] \tag{58}$$

If equilibrium binding and unbinding are fast enough, i.e, $k_b, k_u >> O, E, \gamma$, we get

$$\langle N \rangle = \frac{Qd}{\gamma(\beta + (1-Q))}, \tag{59}$$

$$\nu = \frac{\langle \delta N^2 \rangle}{\langle N \rangle} = 1 + Q\left[(1-Q) - \frac{(\gamma/d+1)\alpha\beta^2}{(\alpha+1)^2}\right]\left[(\beta+1-Q)\left(\beta+1-Q+\beta\frac{\gamma}{d}+\frac{(1+d/\gamma)(\gamma/d)^2\alpha\beta^2}{(\alpha+1)^2}\right)\right]^{-1}$$

(60)

with $\beta = (\tau_O + \tau_E)/\tau_{dead} = d(1/O' + 1/E) = (d/E)[(\alpha+1)/\alpha]$. Equation (60) is shown in figure 4(b).
